# Visual Data Mining of Biological Networks: One Size Does Not Fit All

**DOI:** 10.1371/journal.pcbi.1002833

**Published:** 2013-01-10

**Authors:** Chiara Pastrello, David Otasek, Kristen Fortney, Giuseppe Agapito, Mario Cannataro, Elize Shirdel, Igor Jurisica

**Affiliations:** 1 CRO Aviano, National Cancer Institute, Aviano, Italy; 2 Ontario Cancer Institute, the Campbell Family Institute for Cancer Research, and Techna Institute, University Health Network, Toronto, Ontario, Canada; 3 Department of Medical and Surgical Sciences, University Magna Græcia, Catanzaro, Italy; 4 Departments of Computer Science and Medical Biophysics, University of Toronto, Toronto, Ontario, Canada; Whitehead Institute, United States of America

## Abstract

High-throughput technologies produce massive amounts of data. However, individual methods yield data specific to the technique used and biological setup. The integration of such diverse data is necessary for the qualitative analysis of information relevant to hypotheses or discoveries. It is often useful to integrate these datasets using pathways and protein interaction networks to get a broader view of the experiment. The resulting network needs to be able to focus on either the large-scale picture or on the more detailed small-scale subsets, depending on the research question and goals. In this tutorial, we illustrate a workflow useful to integrate, analyze, and visualize data from different sources, and highlight important features of tools to support such analyses.

## Introduction

For a diverse set of biological problems, network visualization can be a powerful approach for data interpretation and analysis. Many different network visualization tools and software packages are freely available to academic researchers; these systems differ greatly in terms of the features and standards they support, and consequently the analyses they enable. Since users of these visualization tools come from a range of backgrounds in biology and bioinformatics, they have distinct skills and expectations. One or many tools may be used to explore or visualize a single network, each with different strengths and weaknesses.

In this tutorial, we give an in-depth tutorial on how to create informative network visualizations from a set of genes using NAViGaTOR 2.3 as our network visualization tool (http://ophid.utoronto.ca/navigator). Our intent is to explore the relationship between genes known to modulate aging and genes commonly mutated in cancer. We demonstrate several ways that complementary data sources (such as therapeutics and microRNAs) and alternate layout strategies can be combined to offer distinct perspectives on the same underlying biological problem. These network visualizations can illuminate surprising new connections, simplify interpretation, and help speed up biological discovery through data-driven hypothesis generation.

## Workflow

### General Considerations for Biological Network Visualization

#### Network construction

Networks are generally represented within visualization applications as annotated graphs. Biological networks are represented as graphs, and defined by a set of vertices (often referred to as nodes) and edges, plus additional annotations that describe the properties of these objects. This model allows the use of many algorithms devoted to graph analysis. Networks can be obtained in several ways, generally beginning with a list of genes of interest (often derived from experimental data) and an interaction file defining global interactions from a larger set. From this point networks can be manually constructed—with the user assigning positions and node description characteristics, they can be automatically generated (for example, random networks), or could be formed by querying public databases (e.g., protein–protein interaction or pathway databases) to assign further node descriptions. To aid data interchange, several community standards, e.g., PSI-MI [1], BioPAX [2], SBML [3], GML, CML, CellML [4], and ASCII TAB delimited file have been defined. Some commonly used databases of interactions include Intact [5], BioGRID [6], HPRD [7], I2D [8], and iRefindex [9]. Some of these have been integrated under the IMEx consortium [10], and are accessible via the PSICQUIC standard [11]. The features of each of these data formats and sources vary, being extremely dependent on their individual purpose. For example, the *C. elegans* interactions in I2D ver. 1.95 (7,060 proteins/nodes and 57,224 interactions/edges) can be represented as a 3MB TAB delimited file, containing only graph structure, protein names, and interaction sources. The same data, represented in PSI-MI format, is structured to define many other features, such as database cross-references and experiment documentation, and requires 56MB. The time to load these different formats in NAViGaTOR is 10 and 54 seconds, respectively, as complex formats require more system resources within the visualization program. File formats must be carefully selected to serve the needs and competencies of the researcher.

Depending on the workflow and features of a program, it may be possible to merge networks and annotations from multiple sources, while considering context and confidence of individual interactions. Thus, tools supporting data interchange formats such as PSI-MI enable this loose program integration.

Due to the ambiguity of terms and many-to-many relationships to IDs, it is important to note that the accuracy and reproducibility of a representation is entirely dependent on the level of care with which its data was documented. Researchers should always document the source of their data, which nomenclature databases (and their versions) were used (UniProt release 2012_07, mirDIP v2.0, DrugBank v2.5, etc.), and what ID mapping methods have been used in instances where different sources were merged.

#### Layout

Network layouts determine the positions of individual nodes in a coordinate space. There are several basic requirements that a good layout should satisfy. It should provide a biologically meaningful representation of the network while allowing “easy” interpretation by the viewer. Edges between nodes of interest should be easy to follow. This can be achieved by efficient use of screen space and minimization of edge crossings, and by optimal use of transparency and other visualization methods. Layouts can be created by manually or algorithmically placing nodes, or by some combination of these two approaches. For algorithmic layouts, scalability can be important, as biological graphs can often grow to encompass tens of thousands of nodes and millions of edges. Several effective algorithms, such as Graph Drawing with Intelligent Placement (GRIP) [12], have been proposed to address this requirement, as well as algorithms that take advantage of multi-core CPUs or GPUs. Even so, large biological graphs are sometimes impossible to lay out coherently even with the best algorithms, resulting in “hairball” layouts.

#### Visualizing node and edge attributes

The annotations contained in a network can be represented using different visual attributes, such as color, size, and shape. Multiple combinations of attributes to visual feature mapping exist, but some are more suitable and easier to interpret. One has to consider continuous versus discrete attributes, dynamic ranges, and number of features and their values that need to be differentiated. The type of annotation and its visual attribute must also be appropriate: node size can effectively represent a numeric value, but might poorly represent different classes of object. For example, expression can be effectively represented by color, but may also be represented as discrete values with node shape. In both examples, using transparency can further improve interpretability of important parts in the network.

#### Analysis

Systematic analysis of protein–protein interactions (PPIs) and other biological networks can uncover biologically relevant information. PPI networks have a strong structure-function relationship [13], which can be used to help interpret integrated data sets [14]–[16]. Beyond the different data that can be integrated to help visualize highly important nodes in the graph, the analysis can also involve graph features like node degree, node centrality, shortest paths, cliques, cycles in directed graphs, etc.

Diverse data sources and visualizations can offer different perspectives on the same biological data. There are many different metrics for evaluating a graph's aesthetics, such as path bend and edge crossing minimization [17]. However, these metrics often give different results when presented with the same graph, which leaves room for subjective decisions about individual layout tasks. Below, we explore alternative visualizations of the relation between aging and cancer to highlight some of the features discussed above.

### Building the Aging-Cancer Network

#### Network construction

First, we converted 65 human aging genes (downloaded from GenAge build 15, http://genomics.senescence.info/genes/) into 112 UniProt IDs and 475 human cancer genes (from the Sanger Cancer Gene Census, http://www.sanger.ac.uk/genetics/CGP/Census/, accessed Feb. 2012) into 857 UniProt IDs (Table S1) using the I2D web interface (http://ophid.utoronto.ca/ophidv2.201/). The mapping between different database IDs is never one-to-one, and this causes the differences in numbers between the genes and the UniProt IDs list. Next, we uploaded this list of 969 IDs into NAViGaTOR and retrieved known, publicly available human PPIs from I2D 1.95 (this step retrieves all connections that include at least one gene from the starting list). Next, we used the NAViGaTOR search function to select only those nodes corresponding to the cancer or aging gene lists (e.g., query proteins), and deleted all other nodes (we only wanted to consider connections between aging and cancer genes in our analysis). At this point, the network comprised 219 nodes and 1,195 edges (data available as a NAViGaTOR XML file in Dataset S1); the default visualization for this network is the infamous “hairball”. Further layout and analysis steps are needed to highlight the interesting features of this network. An interactive combination of analysis and visualization implements a visual data mining workflow that enables the best integration of user's needs and expertise.

#### Visualizing attributes

**Figure 1:**
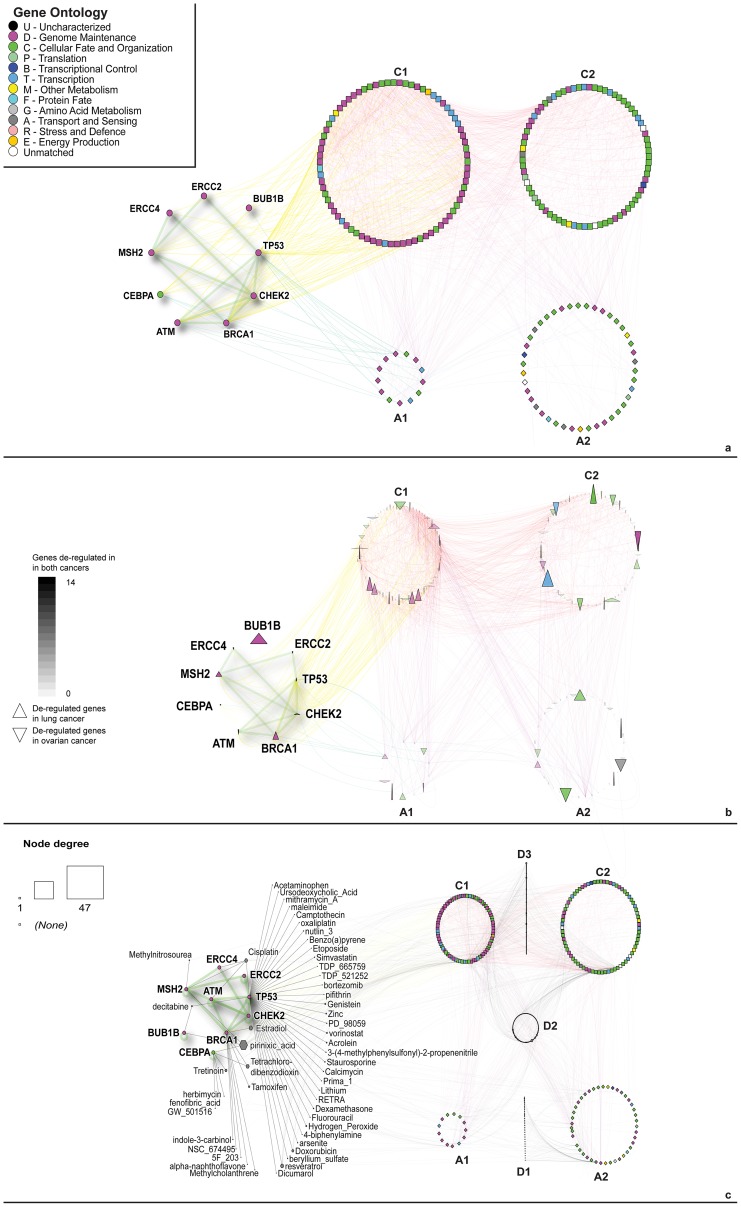
Network visualization of the query genes and their involvement in other tumor types. a) Network built on aging and cancer genes. Labeled nodes belong to both gene lists. Square nodes represent cancer genes while diamonds represent aging genes. b) Deregulation of the network genes in lung (represented by down arrows) and ovarian cancer (represented by up arrows). The height and width of the nodes are proportional to the number of studies where the genes are deregulated. Node transparency corresponds to overall number of studies where the gene is deregulated. c) Network integrating chemical compounds targeting the query genes. Hexagonal nodes represent drugs. The names of the drugs interacting with the shared genes are shown. C: cancer genes, A: aging genes, D: drugs. C1, A1: genes interacting with shared ones, C2, A2: genes not interacting with the shared ones. D1: drugs targeting only aging genes, D2: drugs targeting both aging and cancer genes, D3: drugs targeting only cancer genes. Node colors represent GO categories as per legend. Edges are colored to differentiate inter- and intra-group interactions.

(1) Using shape to distinguish node types. First, we defined two subsets in NAViGaTOR: aging proteins and cancer proteins. We used the set operation tool to create a third subset, defined as the intersection of the first two subsets, i.e., proteins known to be involved both in aging and in cancer. A different node shape has been manually assigned to each subset (Figure 1a and Figure S1). The group of shared genes comprised *ATM, BRCA1, BUB1B, CEBPA, ERCC2, ERCC4, MSH2, TP53*, and *CHEK2*. (2) Using node color to distinguish protein function. Using the I2D plugin, we downloaded the Gene Ontology (GO) [18] terms, PubMed references, and gene synonyms associated with each node. We decided to color the nodes according to the GO category to which they belong (Figure 1a), and to show the gene names of the nodes shared between the aging and cancer lists. We decided to display gene names because they are more interpretable by humans than database IDs, but any text feature associated to the nodes can be displayed as a label. Importantly, gene or protein IDs are best used to interrogate external databases, as they eliminate the ambiguity of name matching.

Pathway enrichment analysis of the proteins in the network using the NAViGaTOR plugin showed that the top two pathways for the complete network as well as for the shared genes are DNA Repair (*p*<2.83^−09^ and *p*<5.65^−06^) and Cell Cycle (*p*<6.15^−09^ and *p*<8.35^−05^) (Figure S2). It has been described that the choice between cell survival and cell death represents the trade-off between cancer and aging, triggered by damage to the DNA [19]. These pathways are highly interconnected [20]; and thus, it is not surprising to identify them to be most represented in our cross-talk genes.

#### Layout

We used a separated circular layout for the nodes shared between the cancer and aging datasets while the other nodes are distributed in circular layouts, including only cancer genes (C) or only aging genes (A) directly interacting with the shared ones (A1, C1) or not (A2, C2). The circular layout lets us appreciate the number of nodes for each category and the distribution of the visual features in the subset. As shown in Figure S3, these views can be further enhanced by sorting the circular view using GO, thus further highlighting the diversity or enrichment of individual GO functions.

### Perspectives and Visualizations of the Aging-Cancer Network: Differentially Regulated Genes in Cancer

We identified network nodes that were significantly differentially regulated in both non-small cell lung and ovarian cancer data, two cancers where incidence rises sharply with age (SEER, http://seer.cancer.gov/).

#### Network construction

We downloaded genes up- or down-regulated in non-small cell lung or ovarian cancer from CDIP, the Cancer Data Integration Portal (http://ophid.utoronto.ca/cdip/), a collection of gene expression data from published studies.

#### Visualizing attributes

To represent up- and down-regulated genes we used node shape. We also related node height to the number of times the gene is aberrantly expressed in lung cancer studies and node width to the number of ovarian cancer studies (Figure 1b and Figure S4). Transparency shows the total number of times the gene was deregulated in the two cancers. Edge color was used to differentiate inter- and intra-group interactions, and node color corresponds to GO biological function.

#### Interpretation

It is now apparent that the shared genes are more frequently aberrantly expressed in ovarian cancer than in non-small cell lung cancer. This implies the involvement of the DNA repair pathways in the two tumor types: they play a central role in ovarian carcinogenesis (all of the high penetrance ovarian cancer susceptibility genes identified so far play a role in DNA repair) [21], while in lung cancer they share causality with signaling pathways (especially those that promote growth) [22].

Network nodes and edges may represent other information besides physical PPIs, e.g., genetic interactions [23], metabolic reactions [24], gene regulations [25], and microRNA–target [26] and drug–target associations [27]. The next paragraph discusses an example. A second one is shown in Figure S5.

### Perspectives and Visualizations of the Aging-Cancer Network: Therapeutics

#### Network construction

We downloaded lists of drugs that target the aging and cancer genes in our network from CTD, the Comparative Toxicogenomics Database (http://ctdbase.org/) [28]. We uploaded these drug–protein interactions into a new subset in NAViGaTOR.

#### Layout

The nodes representing the drugs are distributed in sets close to the nodes category they interact with. For drugs interacting with the shared genes, we used a linear layout to show node names more clearly (Figure 1c and Figure S6).

#### Interpretation

It is interesting to note that 99 compounds (23% of all compounds) target genes from both the cancer and aging subsets. The most connected node in this subset is pirinixic acid, a peroxisome proliferator activated receptor α (PPARα) agonist. PPARα and the genes under its control play a role in the evolution of oxidative stress excesses observed in aging, the activation of the receptor being involved in the restoration of the cellular redox balance [29]. Moreover, it has been demonstrated that several genes involved in cell cycle control and DNA repair pathways are induced upon activation of PPARα [30].

### Finishing Up: Data Interchange and Export

Exporting network data, much like the data import stage of network construction, is possible with many formats, each with their own strengths and weaknesses, such as richness of representation language, speed of loading the file, memory requirements, etc. The selection of an export format depends on the intended recipient of the data and their needs (http://www.cs.utoronto.ca/~juris/data/BI09/).

Analogously to data export, multiple image export formats can be used by individual tools. For networks, it is most useful to extend the standard image formats, e.g., JPEG, TIFF, PNG, BMP, with scalable vector graphics (SVG, http://www.w3.org/Graphics/SVG/) format, which is an XML-based file format for describing two-dimensional vector graphics. SVG format is especially useful for preparing the final annotated figures without loss of quality in Inkscape (http://www.inkscape.org/) or Adobe Illustrator.

In our case, the network files were exported in SVG format and finalized using Adobe Illustrator. The resulting figures are the ones shown in this paper. The original networks in NAViGaTOR XML format are downloadable from the supplementary data (Datasets S2Table S1, S3, S4, S5, S6, S7).

## Conclusions

After discussing the basic network visualization workflow, we demonstrated how the analysis of networks built on PPIs can discover and highlight important features of some genes of interest (in our case, the connection between cancer and aging genes). We also introduced some of the many different kinds of data that are now available to enrich the analysis of a simple gene list (or list of proteins, microRNAs, etc.). While we focused on gene expression, drug targeting, and microRNA involvement with the genes in our network, we highlighted the main principles and steps needed for such integrative network analysis. We discussed node layout and showed how to set up their appearance in some of the many possible ways to give an idea of how networks can effectively visualize large amounts of data by emphasizing differences between subsets and ranking nodes according to a given filter.

Understandably, presented combinations of analysis methods are only a few out of many possible approaches to visual data mining. NAViGaTOR and other similar tools offer the possibility to combine different analyses and data in multiple ways, and the researcher's purpose and interest will determine which workflow is more effective for her data. A comparison of the main features of several well-known network visualization tools is shown in Table S2, and links to a larger number of tools are included as Table S3. Tools that support interactive data visualization enable a useful blend of automation and expert knowledge to customize the workflows and analyses on the fly.

Individual tools need a good balance of performance and useful features. The features needed to complete a task are highly dependent on the available data and the researcher's competencies. There is no single solution applicable to all research scenarios; this requires the constant development of new tools and analysis methods, like the development of plugins to semi-automatically query and integrate data from all possible useful database, and places a responsibility upon the researcher to be aware of and competent in tools that support her needs.

## Supporting Information

Dataset S1 **Hairball.** XML file with only the original data (“hairball”).
(XML)

Dataset S2 **Cancer and aging genes network.** XML file corresponding to Figure S1.
(XML)

Dataset S3 **Pathway network.** XML file corresponding to Figure S2.
(XML)

Dataset S4 **Gene Ontology network.** XML file corresponding to Figure S3.
(XML)

Dataset S5 **Deregulated genes network.** XML file corresponding to Figure S4.
(XML)

Dataset S6 **microRNA network.** XML file corresponding to Figure S5.
(XML)

Dataset S7 **Drugs network.** XML file corresponding to Figure S6.
(XML)

Figure S1 **Cancer and aging genes network.** Labeled nodes belong to both gene lists. Square nodes represent cancer genes while diamonds represent aging genes. C: cancer genes, A: aging genes. C1, A1: genes interacting with the shared genes, C2, A2: genes not interacting with the shared genes. Node colors represent GO categories as per legend. Edges are colored to differentiate inter- and intra-group interactions.
(TIF)

Figure S2 **Pathway network.** Pathway enrichment analysis. Nodes belonging to the most significant pathways are highlighted (DNA Repair *p* = 2.83^−09^ and Cell Cycle *p* = 6.15^−09^; Hypergeometric test). Green highlight: Cell Cycle. Blue highlight: DNA Repair. Red highlight: overlap. C: cancer genes, A: aging genes. C1, A1: genes interacting with the shared genes, C2, A2: genes not interacting with the shared genes. Node colors represent GO categories as per legend. Edges are colored to differentiate inter- and intra-group interactions.
(TIF)

Figure S3 **Gene Ontology network.** Organization of nodes according to their Gene Ontology terms. C: cancer genes, A: aging genes. C1, A1: genes interacting with shared ones, C2, A2: genes not interacting with the shared ones. Node colors represent GO categories as per legend. Edges are colored to differentiate inter- and intra-group interactions.
(TIF)

Figure S4 **Deregulated genes network.** Analysis of the deregulation of the network genes in lung (represented by down triangles) and ovarian cancer (represented by up triangles). The height and width of the nodes are proportional to the number of studies where the genes are deregulated. Transparency represents total number of studies where the gene is deregulated. C: cancer genes, A: aging genes. C1, A1: genes interacting with the shared genes, C2, A2: genes not interacting with the shared genes. Node colors represent GO categories as per legend. Edges are colored to differentiate inter- and intra-group interactions.
(TIF)

Figure S5 **microRNA network.** Integration of microRNAs targeting the original genes. After downloading predicted miRNA–gene interactions for the genes in our network from the mirDIP database ver. 1 (http://ophid.utoronto.ca/mirDIP/), which integrates 12 microRNA prediction datasets, we kept only those interactions that were identified in at least three independent datasets. We analyzed the microRNAs to separate universal microRNAs from pathway-specific ones [25]. Notably, hsa-miR-548c-3p is predicted to target the largest number of shared genes. A recent study implicated this microRNA in the regulation of HMGA1 [31], a proto-oncogene that influences nuclear functions, essential mitochondrial DNA maintenance, and organelle functions. HMGA1 proteins are often over-expressed in cancer cells and alterations to mitochondrial function are frequently present. These two characteristics are also associated with multiple non-cancer diseases and aging [32]. Pathway-specific microRNAs are shown in blue while universal ones are shown in red. C: cancer genes, A: aging genes. C1, A1: genes interacting with the shared genes, C2, A2: genes not interacting with the shared genes. Node colors represent GO categories as per legend. Edges are colored to differentiate inter- and intra-group interactions.
(TIF)

Figure S6 **Drugs network.** Integration of chemical compounds targeting genes in the cancer and aging lists. Octagonal nodes represent drugs. Drug node size corresponds to node degree. The names of the drugs interacting with the shared genes are shown. C: cancer genes, A: aging genes, D: drugs. C1, A1: genes interacting with the shared genes, C2, A2: genes not interacting with the shared genes. D1: drugs targeting only aging genes, D2: drugs targeting both aging and cancer genes, D3: drugs targeting only cancer genes. Node colors represent GO categories as per legend. Edges are colored to differentiate inter- and intra-group interactions.
(TIF)

Table S1 **Lists of genes used for the analysis.**
(XLS)

Table S2 **Comparison of the main features of some network visualization tools.** The table shows a synthetic comparison of the main features of some well-known network visualization tools. ✓ = the tool fully supports the function. ^*^ **L** = the tool can retrieve information stored locally; **R** = capability to retrieve information from remote databases. ^#^ **A** = the tool automatically can get additional information from the selected data sources; **M** = this function is supported only manually. ^$^ **All** = Win, Mac, Linux, **JVM** = Java virtual machine. ^∧^ **S** = Simple, **C** = Complex, and **L** = Limited, refer to the complexity of the analysis supported by each tool.
(DOCX)

Table S3 **List of known protein interaction network visualization tools.**
(DOC)
