# Supplementary figures and images for: Visual Data Mining of Biological Networks: One Size Does Not Fit All
Source: PLoS Comput Biol. 2013 Jan 10;9(1):e1002833. doi: 10.1371/journal.pcbi.1002833 (PMC3547662; doi:10.1371/journal.pcbi.1002833)

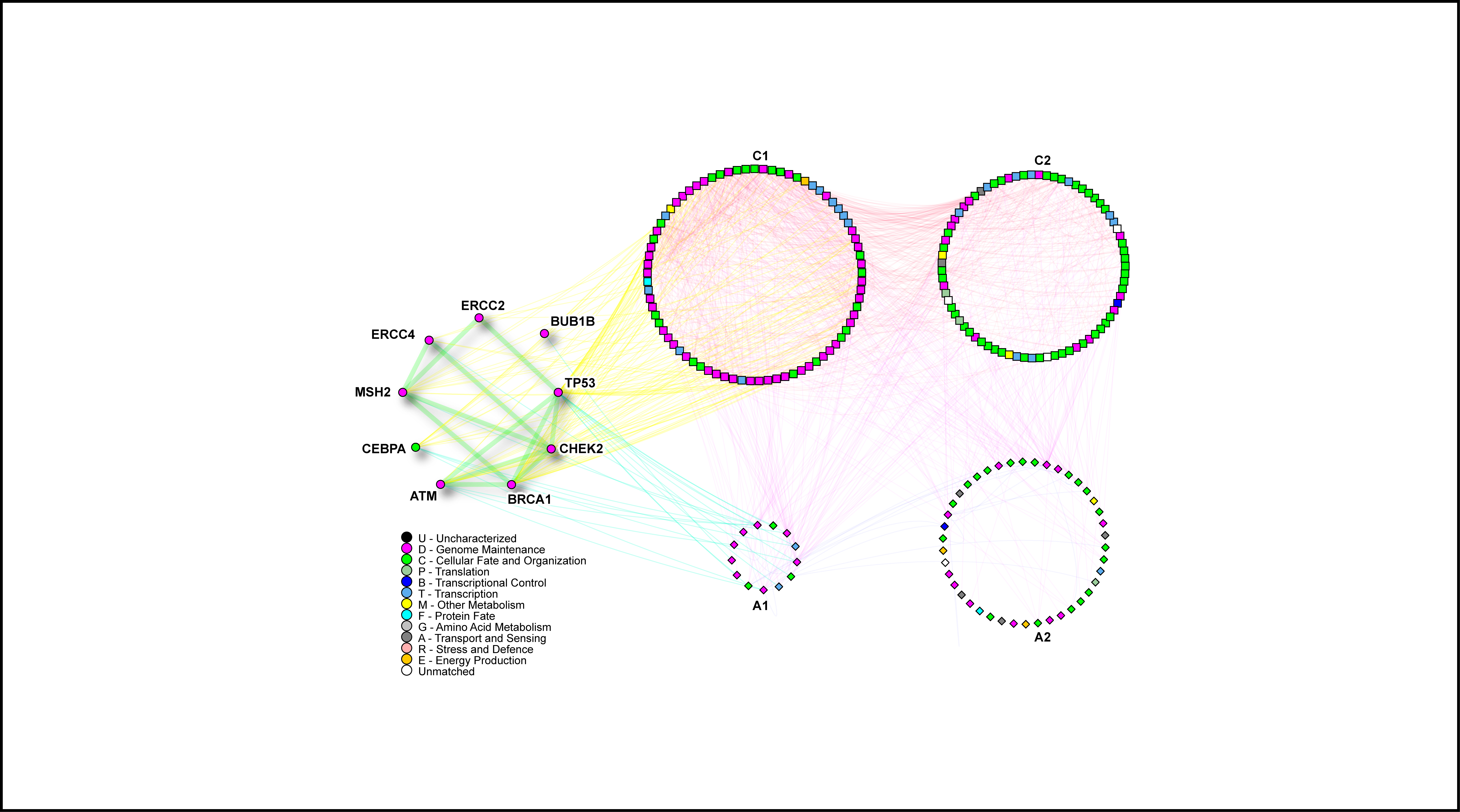

Supplement: Figure S1 — Cancer and aging genes network. Labeled nodes belong to both gene lists. Square nodes represent cancer genes while diamonds represent aging genes. C: cancer genes, A: aging genes. C1, A1: genes interacting with the shared genes, C2, A2: genes not interacting with the shared genes. Node colors represent GO categories as per legend. Edges are colored to differentiate inter- and intra-group interactions. (TIF) [file pcbi.1002833.s008.tif]

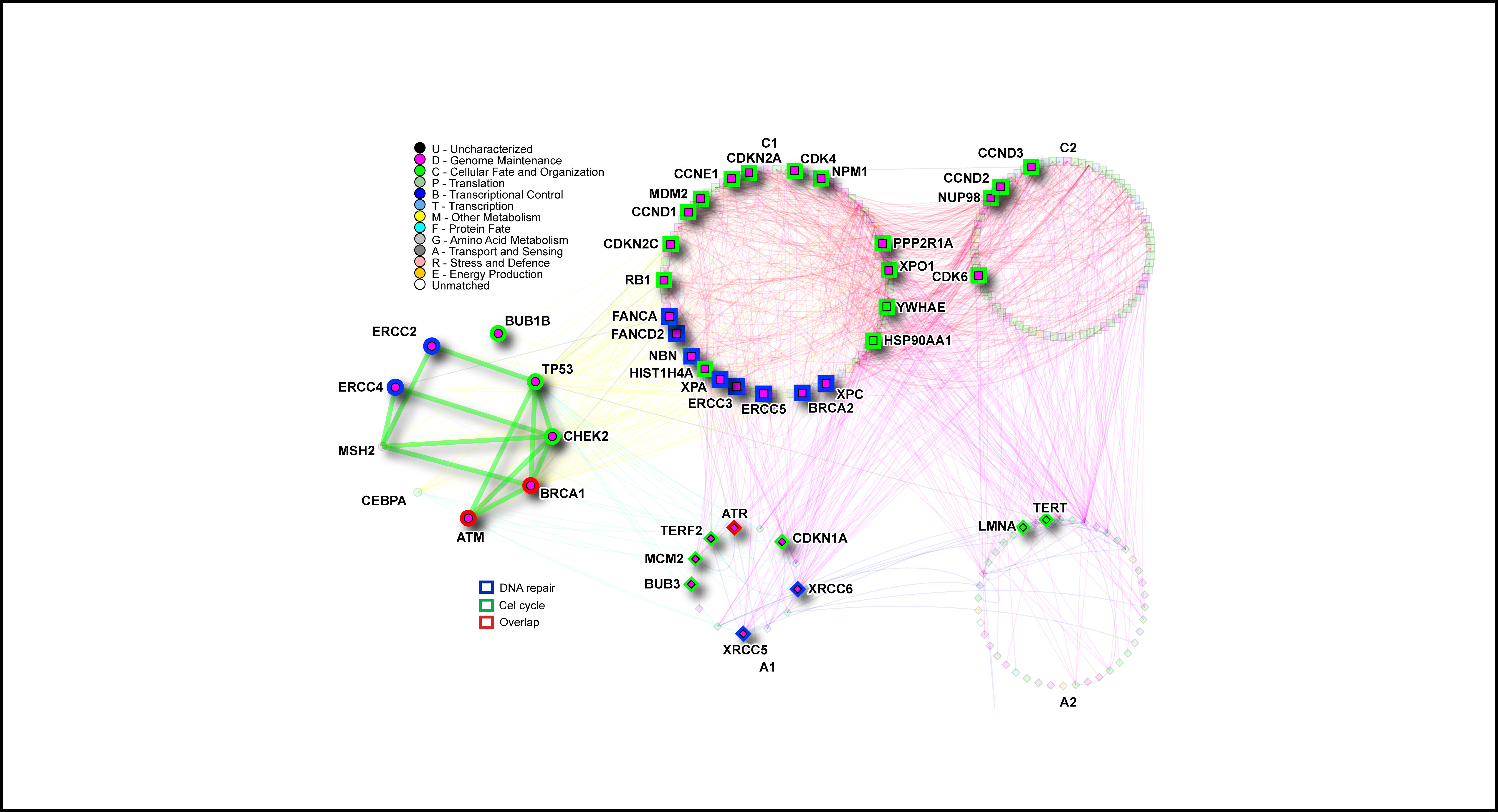

Supplement: Figure S2 — Pathway network. Pathway enrichment analysis. Nodes belonging to the most significant pathways are highlighted (DNA Repair p = 2.83−09 and Cell Cycle p = 6.15−09; Hypergeometric test). Green highlight: Cell Cycle. Blue highlight: DNA Repair. Red highlight: overlap. C: cancer genes, A: aging genes. C1, A1: genes interacting with the shared genes, C2, A2: genes not interacting with the shared genes. Node colors represent GO categories as per legend. Edges are colored to differentiate inter- and intra-group interactions. (TIF) [file pcbi.1002833.s009.tif]

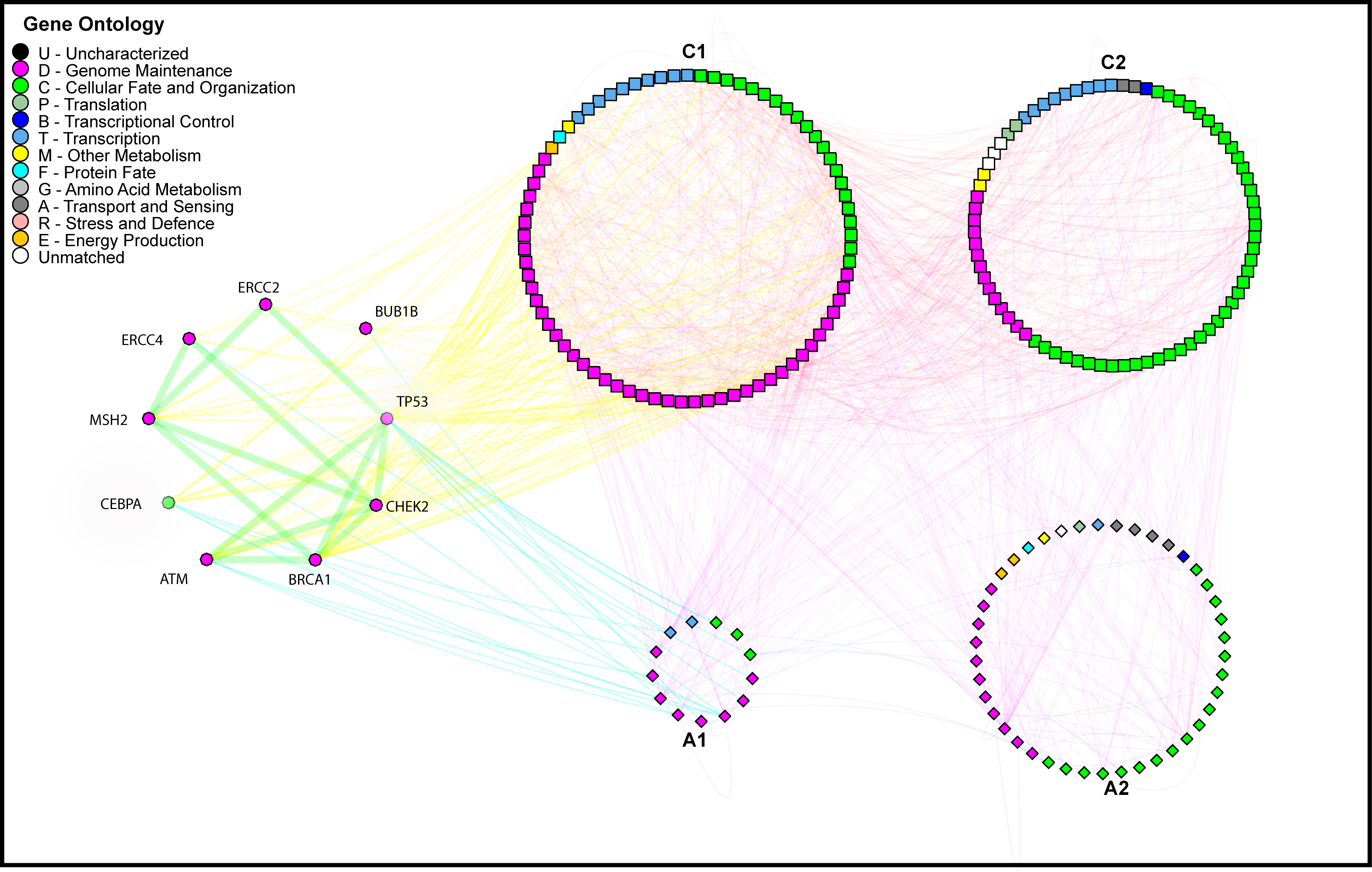

Supplement: Figure S3 — Gene Ontology network. Organization of nodes according to their Gene Ontology terms. C: cancer genes, A: aging genes. C1, A1: genes interacting with shared ones, C2, A2: genes not interacting with the shared ones. Node colors represent GO categories as per legend. Edges are colored to differentiate inter- and intra-group interactions. (TIF) [file pcbi.1002833.s010.tif]

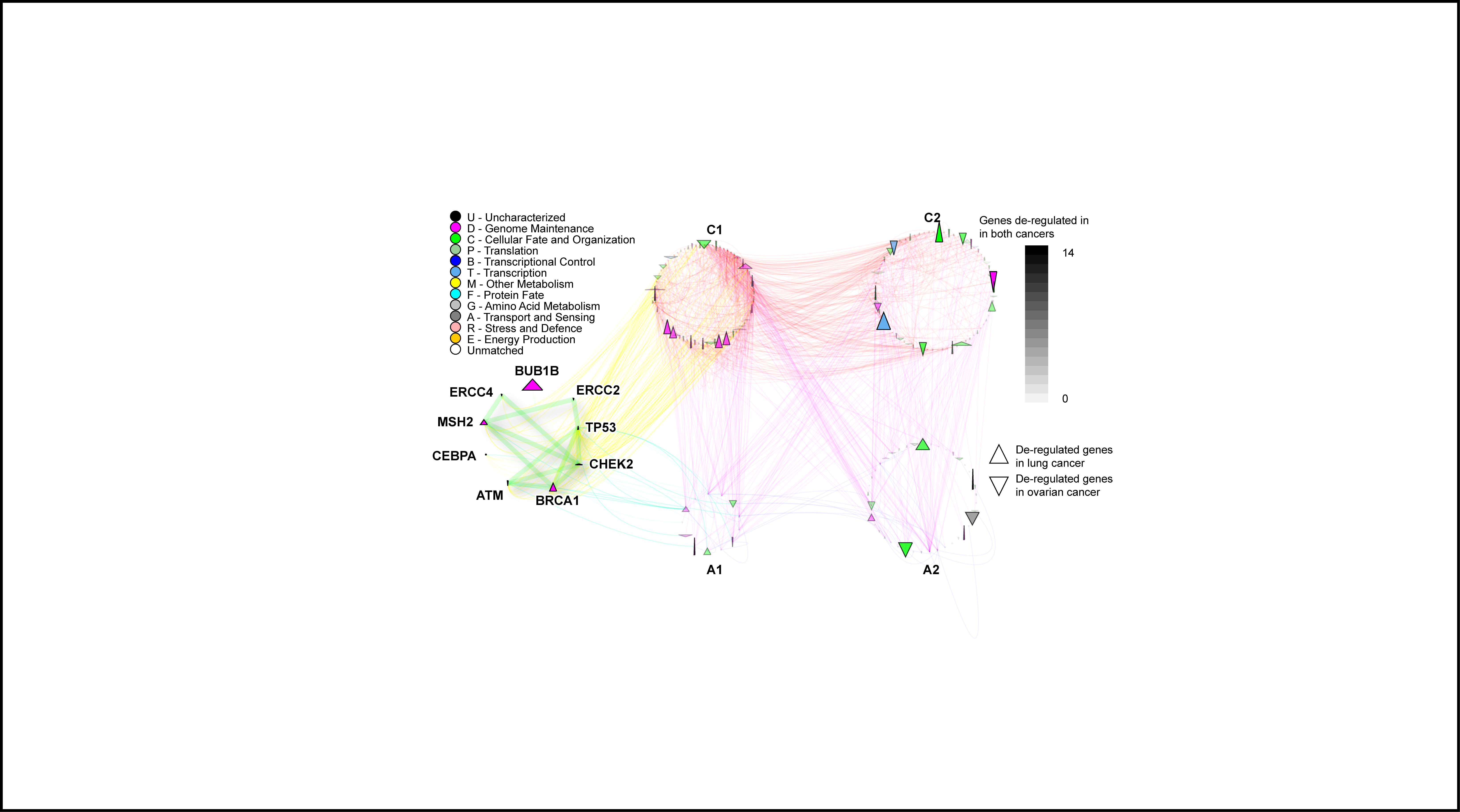

Supplement: Figure S4 — Deregulated genes network. Analysis of the deregulation of the network genes in lung (represented by down triangles) and ovarian cancer (represented by up triangles). The height and width of the nodes are proportional to the number of studies where the genes are deregulated. Transparency represents total number of studies where the gene is deregulated. C: cancer genes, A: aging genes. C1, A1: genes interacting with the shared genes, C2, A2: genes not interacting with the shared genes. Node colors represent GO categories as per legend. Edges are colored to differentiate inter- and intra-group interactions. (TIF) [file pcbi.1002833.s011.tif]

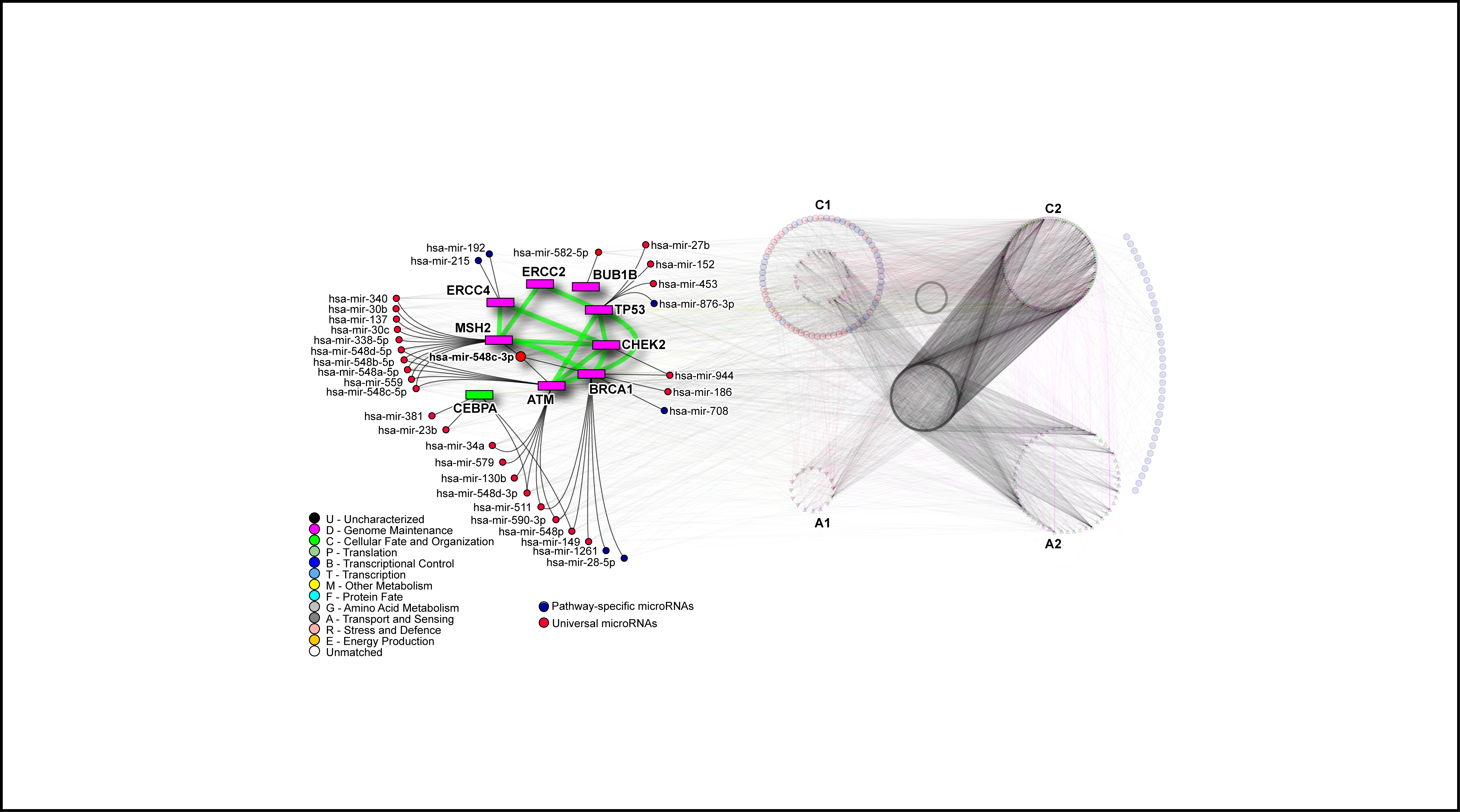

Supplement: Figure S5 — microRNA network. Integration of microRNAs targeting the original genes. After downloading predicted miRNA–gene interactions for the genes in our network from the mirDIP database ver. 1 (http://ophid.utoronto.ca/mirDIP/), which integrates 12 microRNA prediction datasets, we kept only those interactions that were identified in at least three independent datasets. We analyzed the microRNAs to separate universal microRNAs from pathway-specific ones [25]. Notably, hsa-miR-548c-3p is predicted to target the largest number of shared genes. A recent study implicated this microRNA in the regulation of HMGA1 [31], a proto-oncogene that influences nuclear functions, essential mitochondrial DNA maintenance, and organelle functions. HMGA1 proteins are often over-expressed in cancer cells and alterations to mitochondrial function are frequently present. These two characteristics are also associated with multiple non-cancer diseases and aging [32]. Pathway-specific microRNAs are shown in blue while universal ones are shown in red. C: cancer genes, A: aging genes. C1, A1: genes interacting with the shared genes, C2, A2: genes not interacting with the shared genes. Node colors represent GO categories as per legend. Edges are colored to differentiate inter- and intra-group interactions. (TIF) [file pcbi.1002833.s012.tif]

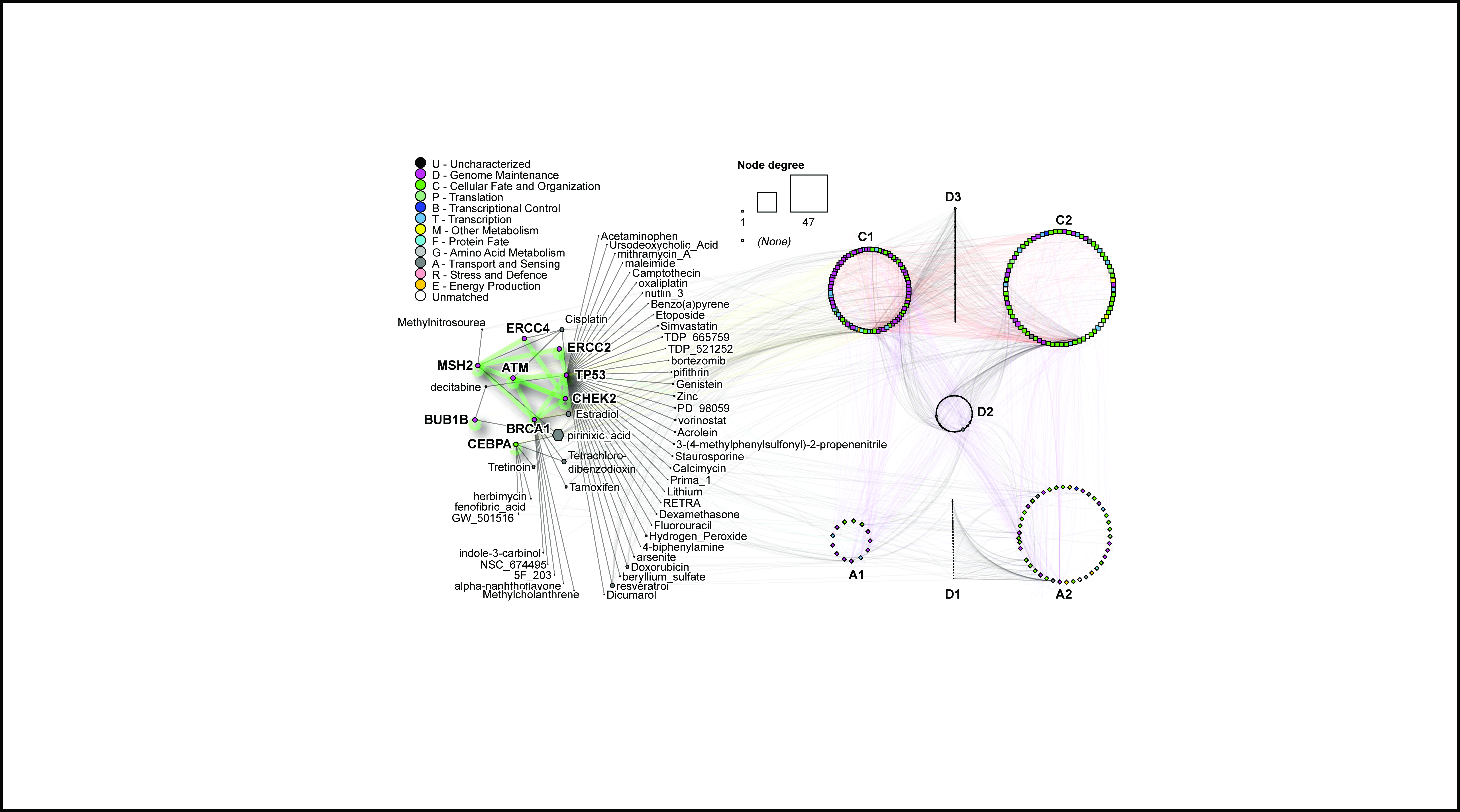

Supplement: Figure S6 — Drugs network. Integration of chemical compounds targeting genes in the cancer and aging lists. Octagonal nodes represent drugs. Drug node size corresponds to node degree. The names of the drugs interacting with the shared genes are shown. C: cancer genes, A: aging genes, D: drugs. C1, A1: genes interacting with the shared genes, C2, A2: genes not interacting with the shared genes. D1: drugs targeting only aging genes, D2: drugs targeting both aging and cancer genes, D3: drugs targeting only cancer genes. Node colors represent GO categories as per legend. Edges are colored to differentiate inter- and intra-group interactions. (TIF) [file pcbi.1002833.s013.tif]
