# Supplementary material for: Visual Data Mining of Biological Networks: One Size Does Not Fit All
Source: PLoS Comput Biol. 2013 Jan 10;9(1):e1002833. doi: 10.1371/journal.pcbi.1002833 (PMC3547662; doi:10.1371/journal.pcbi.1002833)
Supplement: Table S2 — Comparison of the main features of some network visualization tools. The table shows a synthetic comparison of the main features of some well-known network visualization tools. ✓ = the tool fully supports the function. * L = the tool can retrieve information stored locally; R = capability to retrieve information from remote databases. # A = the tool automatically can get additional information from the selected data sources; M = this function is supported only manually. $ All = Win, Mac, Linux, JVM = Java virtual machine. ∧ S = Simple, C = Complex, and L = Limited, refer to the complexity of the analysis supported by each tool. (DOCX) [file pcbi.1002833.s015.docx]

| **Tool** | **URL** | **Plugins**  **Extensibility** | **3D**  **Rendering** | **Database**  **Querying^*^** | **Parallelism**  **Capability** | **Network**  **Annotation^#^** | **Network file**  **import formats** | **Network file**  **export formats** | **Supported Operative System^$^** | **Data**  **Integration** | **Network**  **Analysis^^^** |
| --- | --- | --- | --- | --- | --- | --- | --- | --- | --- | --- | --- |
| **Arena3D** | <http://www.arena3d.org/> | *-* | *-* | *L/R* | *-* | *-* | *PSI-MI, SBML* | *jpeg, net, txt* | All - JVM | *-* | *S* |
| **AVIS** | <http://actin.pharm.mssm.edu/AVIS2/> | *-* | *-* | *R* | *-* | *A* | *avis, BioPax, net, PSI-MI, SBML* | *GIF* | Web-based | *-* | *-* |
| **BioLayout3D** | <http://www.biolayout.org/> | *-* | *✔* | *-* | *✔* | *M* | *dat, gl, graphml, expression, layout, mepn, osp, PSI-MI, SIF, sig, txt, xls, xml* | *jpeg, net, png, txt* | All - JVM | *-* | *S* |
| **Cytoscape** | <http://www.cytoscape.org/> | *✔* | *-* | *L/R* | *✔* | *A* | *BioPAX, gml, graphml, PSI-MI, SBML, SIF, txt, XGMML, xml* | *bmp, eps, GML, jpeg, net, png, pdf, SIF, svg, tiff, txt, XGMML, VizMap* | All - JVM | *✔* | *C* |
| **Medusa** | <http://coot.embl.de/medusa/> | *-* | *-* | *R* | *-* | *A* | *txt* | *eps, jpeg, net, html, png, txt* | All - JVM | *-* | *L* |
| **NAViGaTOR** | <http://ophid.utoronto.ca/navigator/> | *✔* | *✔* | *L/R* | *✔* | *A* | *BioPAX, gml, PSI-MI, txt, xml* | *bmp, jpeg, eps, GML, net, png, pdf, svg, tiff, txt, xml* | All - JVM | *✔* | *C* |
| **ONDEX** | <http://www.ondex.org/> | *✔* | *-* | *L/R* | *-* | *M* | *oxl, net, nwb, SBML* | *SIF, txt, XGMML* | All - JVM | *✔* | *S* |
| **Osprey** | <http://biodata.mshri.on.ca/osprey/servlet/Index> | *-* | *-* | *L/R* | *-* | *A* | *gl, osp, txt* | *jpeg, png, svg, txt* | All - JVM | *-* | *L* |
| **Pajek** | <http://pajek.imfm.si/> | *-* | *-* | *-* | *-* | *M* | *dat, net, paj, txt* | *bmp, eps, net, svg, VizMap* | Win, via wine others | *-* | *S* |
| **VisANT** | <http://visant.bu.edu> | *✔* | *✔* | *R* | *-* | *A* | *BioPAX, txt, xml* | *jpg, owl, png, svg, txt, xml* | All - JVM | *✔* | *C* |
