# Supplementary material for: Visual Data Mining of Biological Networks: One Size Does Not Fit All
Source: PLoS Comput Biol. 2013 Jan 10;9(1):e1002833. doi: 10.1371/journal.pcbi.1002833 (PMC3547662; doi:10.1371/journal.pcbi.1002833)
Supplement: Table S3 — List of known protein interaction network visualization tools. (DOC) [file pcbi.1002833.s016.doc]

**Supplementary Table 3**

| Tool | url |
| --- | --- |
| Arena 3D | <http://www.arena3d.org/> |
| AVIS | <http://actin.pharm.mssm.edu/AVIS2/> |
| BiNA | <http://www.bnplusplus.org/bina/> |
| BiNoM-Biological Network Manager | <http://bioinfo.curie.fr/projects/binom> |
| BioGraphE | <https://www.biopilot.org/docs/ModelAbstraction/NetworksAndPathways/BioGraphE.php> |
| BioLayout Express 3D | <http://www.biolayout.org/> |
| Biological Networks | [http://biologicalnetworks.net](http://biologicalnetworks.net/) |
| cPATH | cbio.mskcc.org/cpath/ |
| Cytoscape | <http://www.cytoscape.org/> |
| GENeVis | <http://www.win.tue.nl/~mwestenb/index.html> |
| KrackPlot | <http://www.andrew.cmu.edu/user/krack/krackplot.shtml> |
| Medusa | <http://coot.embl.de/medusa/> |
| N-Browse | <http://www.gnetbrowse.org/> |
| NAViGaTOR | <http://ophid.utoronto.ca/navigator/> |
| Negopy | <http://www.sfu.ca/personal/archives/richards/Pages/negopy.htm> |
| Network Workbench | [http://nwb.cns.iu.edu](http://nwb.cns.iu.edu/) |
| ONDEX | <http://www.ondex.org/verify.php> |
| Osprey | <http://biodata.mshri.on.ca/osprey/servlet/Index> |
| Otter | <http://www.caida.org/tools/visualization/otter/> |
| Pajek | <http://pajek.imfm.si/> |
| PATIKA | [http://www.patika.org](http://www.patika.org/) |
| PIVOT | <http://acgt.cs.tau.ac.il/pivot/> |
| PopTools | [http://www.poptools.org](http://www.poptools.org/) |
| ProViz | <http://cbi.labri.fr/eng/proviz.htm> |
| SimWiz | <http://projects.villa-bosch.de/bcb/software/software/Ulla/SimWiz/> |
| SNAVY | <http://code.google.com/p/snavi/> |
| Snazer | <https://www.cosbi.eu/index.php/research/prototypes/snazer> |
| SpectralNET | <http://www.broadinstitute.org/science/programs/chemical-biology/spectralnet> |
| Tulip | <http://sourceforge.net/projects/auber/> |
| VANLO | <http://stubber.math-inf.uni-greifswald.de/VANLO/> |
| VANTED | <http://sourceforge.net/projects/vanted/> |
| VisANT | [http://visant.bu.edu](http://visant.bu.edu/) |
| yEd | <http://www.yworks.com/en/products_yed_about.html> |
